# Clinical characteristics and survival analysis of Chinese ovarian cancer patients with RAD51D germline mutations

**DOI:** 10.1186/s12885-022-10456-z

**Published:** 2022-12-21

**Authors:** Hongwen Yao, Ning Li, Hua Yuan

**Affiliations:** Department of Gynecologic Oncology, National Cancer Center/National Clinical Research Center for Cancer/Cancer Hospital, Chinese Academy of Medical Sciences and Peking Union Medical College, Beijing, 100021 China

**Keywords:** RAD51D, Germline mutations, Ovarian tumor, Clinical characteristics, Survival.

## Abstract

**Objectives:**

We aimed to describe the behavior among Chinese ovarian cancer patients with RAD51D germline mutations at our institution.

**Methods:**

Next-generation sequencing (NGS) was conducted for the entire coding regions and exon/intron boundaries of the RAD51D genes in 781 Chinese ovarian cancer patients treated at our institution from January 1, 2015 to August 1, 2021. Clinicopathological characteristics, treatment modalities, and outcomes were assessed for ovarian cancer patients with RAD51D germline mutations.

**Results:**

RAD51D germline pathogenic mutations were detected in 1.7% (13/781) of patients in this cohort. RAD51D c. 270_271dup (p. Lys91fs) mutation was the most common mutation which was found in 7 patients (7/13, 53.1%). Patients median age at diagnosis was 58 years (range: 45–69 years). 46.2% (6/13) of them were diagnosed after 60 years. Only 1 patient (1/13, 7.7%) had a family history of ovarian or breast cancer. And 1 patient (1/13, 7.7%) had a personal history of breast cancer. The FIGO 2014 distribution by stage was: stage II in 1 patient (7.7%), stage III in 9 patients (69.2%) and stage IV in 3 patient (23.1%). 92.3% (12/13) patients had high-grade serous carcinoma. 2 patients (2/13, 15.4%) had a primary peritoneal cancer. The majority of patients in the entire cohort were reported to be platinum sensitive (92.3%, 12/13) with a platinum-free interval (PFI) of > 6 months.

For patients who received PARPis for 2nd line maintenance treatment (*n* = 5), 2 patients discontinued PARPis treatment after 33.5 and 8.1 months of duration. Other 3 patients are still on therapy with a duration of 2.4, 13.8 and 30.1 months at the date of data cutoff. 1 patient received PARPi as salvage treatment with a duration of only 1.2 months.

Nine patients (9/13, 69.2%) relapsed during follow up and all of them relapsed within 2 years after diagnosis, among which 88.9% (8/9) were classified as platinum-sensitive recurrence (PSR), and only 1 patient was classified as platinum-resistant recurrence (PRR). Median PFS for the entire cohort was 17.3 months. Median PFS for the PSR subgroup was 15.9 months. 2 patients died during follow-up. The OS of these 2 patients was 17.2 and 39.6 months. The 5-year OS rate was 67.5%.

**Conclusions:**

RAD51D germline mutations are more frequent in Chinese ovarian cancer patients than other population. Few patients have a family history of ovarian or breast cancer, and personal history of breast cancer. Most patients are diagnosed after 50 years. The sensitivity to PARP inhibitors of patients with RAD51D germline mutations need a further analysis.

## Introduction

Epithelial ovarian cancer (EOC) is a major cause of death in women worldwide [1]. In 2016, there were approximately 57,200 new cases of ovarian cancer diagnosed and 27,200 ovarian cancer deaths in China [2].

RAD51 paralogs (RAD51B, RAD51C, RAD51D, XRCC2, and XRCC3) have recently been involved in breast and ovarian cancer predisposition [3]. RAD51 family members are known to be involved in the homologous recombination and repair of DNA. RAD51D is an ovarian cancer predisposition gene. The frequencies of RAD51D germline pathogenic variants varies from 0.2 to 0.57% in EOC [4–13]. RAD51D has a mutation frequency of 1.8% (5/273) in EOC in China [14].

RAD51D is a moderate ovarian cancer susceptibility gene [5]. A recent meta-analysis of approximately 23,000 ovarian cancers ranked RAD51D as a relatively high cancer risk gene [13]. The estimated cumulative risks of developing tubo-ovarian carcinoma and breast cancer to age 80 years were 13% (95% CI: 7–23%) and 20% (95% CI: 14–28%) for RAD51D pathogenic variant carriers [12]. RAD51D mutation testing may have clinical utility in individuals with ovarian cancer and their families [4]. RAD51D analysis should be implemented into the multi-gene testing for high-risk ovarian cancer patients [15, 16].

RAD51D involves in homologous recombination (HR) in the repair of double-strand breaks. The presence of germline and somatic HR mutations was highly predictive of primary platinum sensitivity and improved survival [17]. In addition, clinical trials have demonstrated promising response rates among patients receiving PARP inhibitors (PARPi), especially for BRCA1 or BRCA2 mutation carriers [18–27]. RAD51C and RAD51D mutations predict response to PARPis, similar to BRCA1/2 mutations [28, 29].

Currently, given the rarity of RAD51D mutations, the clinical characteristics and survival of RAD51D germline mutations carriers are not fully elucidated in Chinese ovarian cancer population. In the current study, we investigated the clinical characteristics and survival of Chinese ovarian cancer patients with RAD51D germline mutations.

## Patients and methods

### Patients

Eligible patients were 18 years of age or older and had diagnosed epithelial ovarian cancer, primary peritoneal cancer, or fallopian-tube cancer. Following Institutional Review Board approval, we performed a retrospective analysis of all patients with RAD51D germline pathogenic mutations from January 1, 2015 to August 1, 2021 who received treatment in the Department of Gynecological Oncology of Cancer Hospital, Chinese Academy of Medical Sciences, National Cancer Center. Only patients with a diagnosis of primary EOC confirmed by an experienced gynecologic pathologist in our hospital were included. The patients’ full medical records were included in this study. Clinical and pathologic variables, treatment modalities, and outcomes were assessed. Stage was retrospectively assigned using the 2014 International Federation of Gynecology and Obstetrics (FIGO) staging classification for cancer of the ovary, fallopian tube, and peritoneum. All methods were performed in accordance with the relevant guidelines and regulations (Declaration of Helsinki).

### Methods

A total of 781 Chinese women with primary ovarian cancer treated at our institution from January 1, 2015 to August 1, 2021 were included in our study. All patients had genomic DNA extracted from blood samples of sufficient quantity and quality for RAD51D mutation analysis. Next-generation sequencing (NGS) was conducted for the entire coding regions and exon/intron boundaries of the RAD51D genes. All the testing was done in the department of pathology of our hospital.

## Statistical analyses

For the survival analyses, progression-free survival (PFS) was defined as the time from the date of diagnosis to the earliest date of disease progression, or death from ovarian cancer without a recorded relapse. Overall survival (OS) was defined as the time from the date of diagnosis to death for which ovarian cancer was the primary or underlying cause. Survival was estimated using the Kaplan–Meier product-limit method, and differences were tested for statistical significance using the log-rank test. Two-sided *P* values less than 0.05 were considered to be statistically significant. All analyses were performed using the SPSS Statistics 20.0 software.

## Results

### RAD51D germline pathogenic mutations

RAD51D germline pathogenic mutations were detected in 1.7% (13/781) of patients in this cohort, of which 7 (53.8%) were frameshift indels, 6 (46.2%) were nonsense substitutions. RAD51D c. 270_271dup (p. Lys91fs) mutation was found in seven patients (7/13, 53.1%; Table 1). c. 556C > T (p. Arg186Ter) mutation was detected in three patients (3/13, 23.1%; Table 1). Other three pathogenic mutations were observed once each in either case (c.574C > T, p.Gln192Ter; c.757C > T, p.Arg253Ter; c.898C > T, p.Arg300Ter; Table 1).

**Table 1:** Clinicopathological characteristics and type of PARPi, therapy duration, reasons for treatment discontinuation among patients who received PARPi treatment

ID	RAD51D mutation	Age	Primary tumor location	Histology	FIGO stage	PFS (months)	Follow-up	PARPi type	PARPi setting	PARPi duration (months)	Reasons for treatment discontinuation
1	c.270_271dup, p.Lys91fs	63	Peritoneal	HGSC	IIIC	14.1	Recurrence	Olaparib	2 L maintenance	2.4	Still on therapy
2	c.270_271dup, p.Lys91fs	62	Peritoneal	HGSC	IIIC	17.4	Recurrence	Olaparib	2 L maintenance	30.1	Still on therapy
3	c.270_271dup, p.Lys91fs	69	Ovarian	HGSC	IIIC	12.8	Recurrence	Niraparib	2 L maintenance	33.5	PD
4	c.270_271dup, p.Lys91fs	62	Ovarian	HGSC	IVB	15.9	Recurrence	Olaparib	2 L maintenance	8.1	PD
5	c.270_271dup, p.Lys91fs	49	Ovarian	HGSC	IIIC	11.4	NED	None			
6	c.270_271dup, p.Lys91fs	66	Ovarian	HGSC	IIIC	12.1	NED	None			
7	c.270_271dup, p.Lys91fs	47	Ovarian	OCCC	IIA	39.3	NED	None			
8	c.556C > T, p.Arg186Ter	66	Ovarian	HGSC	IVB	17.4	Recurrence	Olaparib	2 L maintenance	13.8	Still on therapy
9	c.556C > T, p.Arg186Ter	45	Ovarian	HGSC	IIIC	13.2	NED	None			
10	c.556C > T, p.Arg186Ter	58	Ovarian	HGSC	IVB	15.9	Recurrence	None			
11	c.574C > T, p.Gln192Ter	54	Ovarian	HGSC	IIIB	9.0	Recurrence	None			
12	c.757C > T, p.Arg253Ter	52	Ovarian	HGSC	IIIC	22.4	Recurrence	None			
13	c.898C > T, p.Arg300Ter	58	Ovarian	HGSC	IIIC	17.3	Recurrence	Olaparib	4 L salvage	1.2	PD

Abbreviations: *FIGO* International Federation of Gynecology and Obstetrics, *HGSC* high-grade serous carcinoma, *NED* no evidence of disease, *PD* progressive disease

### Clinicopathological characteristics

13 patients who carried RAD51D germline mutations came from 13 unrelated families. Patients median age at diagnosis was 58 years (range: 45–69 years). 46.2% (6/13) of them were diagnosed after 60 years. Only 1 patient (1/13, 7.7%) had a family history of ovarian or breast cancer. And 1 patient (1/13, 7.7%) had a personal history of breast cancer. Other 11 patients (84.6%) had no family history of ovarian or breast cancer, and no personal history of breast cancer. Four patients (30.8%) had a family history of pancreatic, prostate, gastric and colorectal cancer in relatives (Table 2).

**Table 2:** Clinicopathological characteristics of patients with RAD51D mutation in the entire cohort

Clinical Characteristics	RAD51D mt carriers	
	***n***	%
**Median age (Range), years**	**58 (45–69)**	
**Age at diagnosis (year)**		
< 50	3	23.1
50–59	4	30.8
≥ 60	6	46.2
**Family history of ovarian or breast cancer**		
Yes	1	7.7
No	12	92.3
**Personal history of breast cancer**		
Yes	1	7.7
No	12	92.3
**Type of surgery**		
Primary cytoreductive surgery	2	15.4
Interval cytoreductive surgery	11	84.6
**FIGO stage**		
I	0	0.0
II	1	7.7
III	9	69.2
IV	3	23.1
**Primary tumor location**		
Ovary	11	84.6
Peritoneal	2	15.4
**Tumor histopathology**		
High-grade serous	12	92.3
Clear cell	1	7.7
**Postoperative residual disease status**		
Optimal (R0/R1)	12	92.3
Suboptimal (≥R2)	1	7.7
**Recurrence**		
Platinum-sensitive recurrence	8	61.5
Platinum-resistant recurrence	1	7.7
None	4	30.8
**PARPi treatment**		
Olaparib	5	38.5
Niraparib	1	7.7
None	7	53.8

Abbreviations: *BMI* body mass index, *FIGO* International Federation of Gynecology and Obstetrics

1 patient with c. 270_271dup mutation was diagnosed with a breast cancer at 56 years of age and an metachronous ovarian cancer at 63 years.

The FIGO 2014 distribution by stage was: stage II in 1 patient (7.7%), stage III in 9 patients (69.2%) and stage IV in 3 patient (23.1%). 92.3% (12/13) patients had high-grade serous carcinoma. Two patients (2/13, 15.4%) had a primary peritoneal cancer (Table 2).

### Treatment

11 patients (11/13, 84.6%) received neoadjuvant chemotherapy. All patients received cytoreduction surgery and 12 patients (12/13, 92.3%) achieved R0/R1 resection. All patients received paclitaxel + carboplatin regimen chemotherapy after cytoreduction surgery. The majority of patients in the entire cohort were reported to be platinum sensitive (92.3%, 12/13) with a platinum-free interval (PFI) of > 6 months after completing first-line therapy.

### PARPis treatment

6 patients (6/13, 46.2%) received poly (adenosine diphosphate [ADP]–ribose) inhibitors (PARPis) treatment. Among these patients (*n* = 6), 83.3% (5/6) patients received olaparib, 16.7% (1/6) patients received niraparib (Table 1). 83.3% (5/6), 16.7% (1/6) patients received PARPis for first relapsed maintenance treatment, and salvage treatment, respectively (Table 1). Three patients discontinued PARPis treatment because of disease progression.

For patients who received PARPis for 2nd line maintenance treatment (*n* = 5), 2 patients discontinued PARPis treatment after 33.5 and 8.1 months of duration. Other 3 patients are still on therapy with a duration of 2.4, 13.8 and 30.1 months at the date of data cutoff. One patient received PARPi as salvage treatment with a duration of only 1.2 months (Table 1).

Two patients received two lines of PARPis treatment. One patient received PARPi1 (niraparib) as 2nd maintenance treatment with a duration of 33.5 months. After PARPi1 progression, the patient received PARPi2 (olaparib ± apatinib) as subsequent salvage treatment with a duration of 33.5 months and discontinued PARPi2 treatment because of the toxicity. The other patient received PARPi1 (olaparib) as 2nd maintenance treatment with a duration of 8.1 months. After PARPi1 progression, the patient received cytoreduction surgery and PARPi2 (fluzoparib) as 3rd maintenance treatment with a duration of 4.9 months and discontinued because of disease progression.

### Survival analysis

**Fig. 1:**
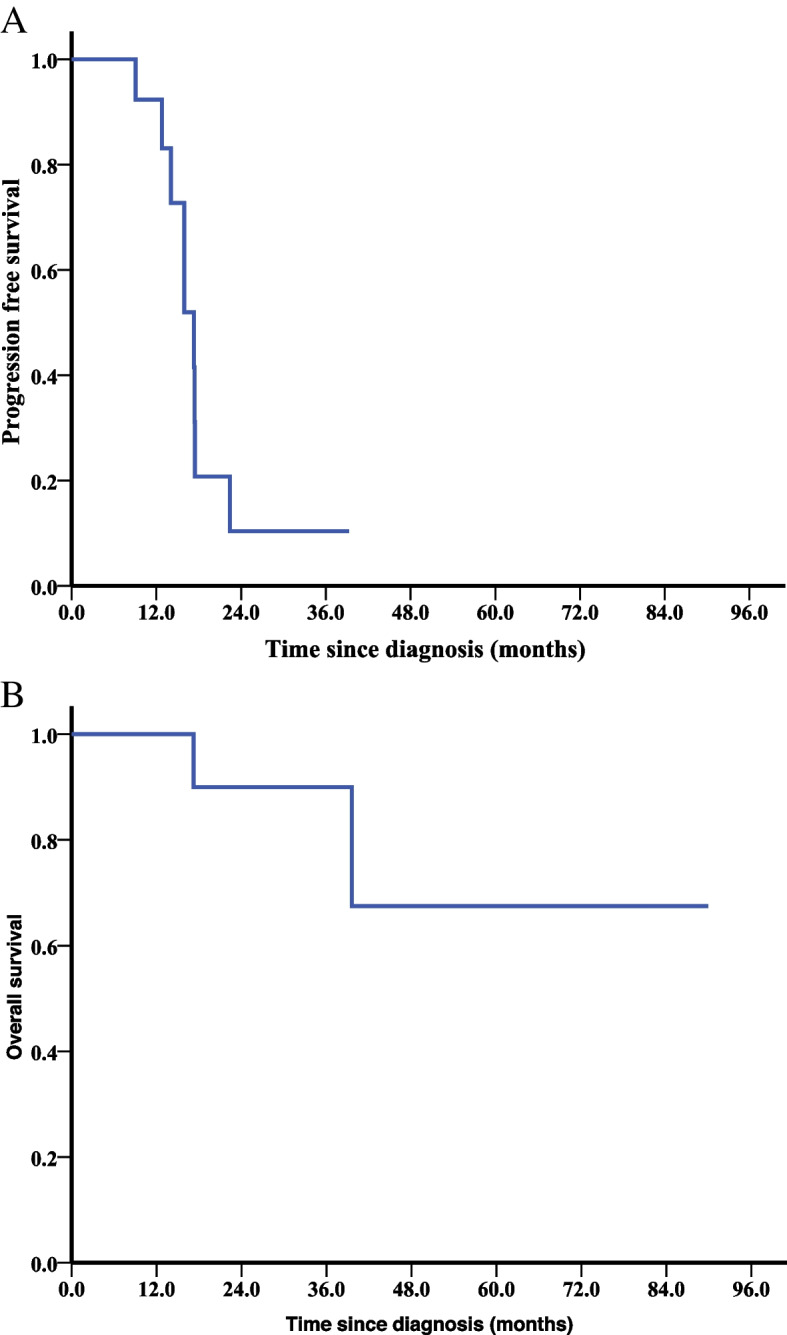
Survival analyses by the Kaplan–Meier method in the entire cohort (*n* = 13). (**A**) Progression-free survival (PFS) and (**B**) overall survival (OS)

The median follow-up was 30.5 months (range: 11.4–89.9 months). Nine patients (9/13, 69.2%) relapsed during follow up and all of them relapsed within 2 years after diagnosis, among which 88.9% (8/9) were classified as platinum-sensitive recurrence (PSR), and only 1 patient was classified as platinum-resistant recurrence (PRR). Median PFS for the entire cohort was 17.3 months (Fig. 1A). Median PFS for the PSR subgroup was 15.9 months.

Two patients died during follow-up. The OS of these 2 patients was 17.2 and 39.6 months. The 5-year OS rate was 67.5% (Fig. 1B).

## Discussion

To our knowledge, the current study is one of the largest studies to report the clinical characteristics and survival of Chinese ovarian cancer patients with RAD51D germline mutations. We found that RAD51D germline mutations are more frequent in Chinese ovarian cancer patients and these patients may derive benefit from PARPis treatment.

RAD51D is a member of the RAD51 protein family which plays an important role in DNA damage repair. The frequencies of RAD51D germline pathogenic mutations varied from 0.2 to 0.57% in EOC [4–13]. The frequency of RAD51D mutations in our study was 1.7% which was higher than that of the reports in other studies. It’s worth noting that the frequency of RAD51D mutations was 0.38% among a large series of unselected breast cancer patients in the Chinese population which was also higher than that of the reports in Caucasian women [30, 31]. The exact role of RAD51D in hereditary breast and ovarian cancer may be different in Chinese and Caucasian women. Previous study showed that RAD51D mutation predispose to ovarian carcinoma but not to breast carcinoma in Caucasian women [32, 33]. RAD51D may play more important role in Chinese hereditary breast and ovarian cancer patients.

In our large cohort of Chinese ovarian cancer patients, RAD51D c.270_271dup was the most frequency pathogenic variant, accounting for 53.8% of all RAD51D pathogenic variants. This variant was observed in 14/18394 chromosomes in the East Asian population according to the Genome Aggregation Database which was rare in Caucasian women [34]. RAD51D c. 270_271dup mutation may be suspected founder mutations in our study. However, owing to the small number of mutation carriers, therefore, further studies involving more mutation carriers are needed to confirm whether these recurrent pathogenic variants are founder or recurrent mutations. The RAD51D c.556C > T recurrent pathogenic variants was found in 3 unrelated cases in our study. This variant was identified in one family previously, where it was found to segregate with the phenotype across 4 family members [4]. The variant was observed in 6/10256 chromosomes in the Ashkenazi Jewish population [34].

Given the rarity of RAD51D mutations, there is limited data about the clinical characteristics and survival of mutation carriers. RAD51D mutation carriers were more likely to suffer from premenopausal ovarian tumors [35]. Song et al. found more mutation carriers were diagnosed at ages 40 to 49 years than noncarriers, and no mutation carrier was diagnosed with ovarian cancer before age 40 years [5]. Our present observations are in agreement with these findings. The median age of ovarian cancer diagnosis in our study was much older than others. Carriers of a mutation in any of the RAD51 genes were more likely than noncarriers to have a family history of ovarian cancer, although this difference was not statistically significant [5]. However, we found only 1 patient (1/13, 7.7%) had a family history of ovarian or breast cancer. We also found several other cancers were present in relatives, such as pancreatic, prostate, gastic and colorectal cancer, which was in accordance with other studies [4].

Emerging evidence showed homologous recombination (HR) is required to maintain genomic integrity in response to DNA-damaging agents and replication stress [36]. Similarly, RAD51D mutation carriers had a better response to first-line platinum chemotherapy. It is worth noting that, all patients who progressed after first line platinum based chemotherapy relapsed within two years after diagnosis and no patients received PARPis treatment as first line maintenance. Cells deficient in RAD51D are sensitive to treatment with a PARPi, suggesting a possible therapeutic approach for cancers arising in RAD51D mutation carriers [4]. Patients with mutations in RAD51C and RAD51D, are extremely vulnerable to PARP inhibition [28, 29]. Our present observation is in agreement with these findings. Investigator-assessed median PFS was (19.1 months [95% CI 16.3–25.7]) and (5.5 months [5.2–5.8]) respectively, for patients who received olaparib or placebo treatment in SOLO2 [26]. In our study, patients who received PARPis for 2nd line maintenance treatment (*n* = 5), 2 patients progressed after 33.5 and 8.1 months of PARPis treatment. Other 3 patients are still on therapy with a duration of 2.4, 13.8 and 30.1 months at the date of data cutoff. We draw a preliminary conclusion that patients with RAD51D germline mutations may derive benefit from PARPis treatment. However, since the mutation spectrum of RAD51D was different in Chinese and Caucasian women, the exact role of RAD51D in hereditary breast and ovarian cancer should be further explored. Kondrashova et al. found that analyses of primary and secondary mutations in RAD51D provide evidence for these primary mutations in conferring PARPis sensitivity and secondary mutations as a mechanism of acquired PARPis resistance [37]. It is important to identify the mechanism of PARPis resistant at the time of subsequent treatment to provide the best therapeutic options going forward in patients with RAD51D mutations.

The age at ovarian cancer diagnosis is older for women with mutations in RAD51D, suggesting that it is safe to delay risk-reducing salpingo-oophorectomy (RRSO) until age 45–50 in RAD51D mutation carriers [38]. The National Comprehensive Cancer Network (NCCN) guidelines for breast and ovarian cancer recommend that RRSO should be considered beginning at age 45 to 50 years in women carriers of germline variants in RAD51D. The median age at diagnosis was 58 years and nearly half of them were diagnosed after 60 years in our study.

There are three limitations to our study. The current study was retrospective, and the primary treatment was not assigned at randomized. All patients in this study came from our single center and the sample size of the patients with RAD51D mutation was relatively small. Therefore, caution is required when interpreting our results. Clinicopathological characteristics, treatment modalities, and outcomes for ovarian cancer patients with RAD51D germline mutations should be studied in prospective manner and further independent studies are warranted to confirm this observation.

## Conclusion

RAD51D germline mutations are more frequent in Chinese ovarian cancer patients than other population. Few patients have a family history of ovarian or breast cancer, and personal history of breast cancer. Most patients are diagnosed after 50 years. The sensitivity to PARP inhibitors of patients with RAD51D germline mutations need a further analysis.

## Data Availability

The datasets generated and/or analysed during the current study are available in the the ClinVar repository (https://www.ncbi.nlm.nih.gov/clinvar/) (Accession numbers: SCV002754422, SCV002754421, SCV002754417, SCV002605543, SCV002605542).

## Abbreviations

NGS: Next-generation sequencing
EOC: Epithelial ovarian cancer
FIGO: International Federation of Gynecology and Obstetrics
PFS: Progression-free survival
OS: Overall survival
PARPis: Poly (adenosine diphosphate [ADP]–ribose) inhibitors
HR: Homologous recombination
RRSO: Risk-reducing salpingo-oophorectomy
NCCN: National Comprehensive Cancer Network
IRB: Institutional Review Board.
